# Toll-like receptor 4 single-nucleotide polymorphisms Asp299Gly and Thr399Ile in ovarian cancers

**DOI:** 10.3892/ol.2014.2113

**Published:** 2014-05-07

**Authors:** AN-CONG WANG, FENG-XIA WU, YONG-SHENG GAO, XIU-GUI SHENG

**Affiliations:** 1 Department of Reproductive Medicine, Linyi People’s Hospital, Linyi, Shandong 276003, P.R. China; 2 Department of Gynecologic Oncology, Shandong Cancer Hospital and Institute, Jinan, Shandong 250117, P.R. China; 3 Department of Anatomy, Shandong University, Jinan, Shandong 250012, P.R. China; 4 Department of Pathology, Shandong Cancer Hospital and Institute, Jinan, Shandong 250117, P.R. China

**Keywords:** toll-like receptor 4, single-nucleotide polymorphism, ovarian cancer

## Abstract

Toll-like receptor (TLR4) 4 is present in numerous cell types and serves as the first point of defense in the innate immune system. Single-nucleotide polymorphisms (SNPs) are present in a number TLR genes and have been associated with various infection and inflammation disorders. Asp299Gly and Thr399Ile, TLR4 SNPs, are associated with tumor progression. In the present study, cases of ovarian cancer were analyzed with regards to Asp299Gly and Thr399Ile of the TLR4 gene. Genotype analysis was performed using DNA from tissue samples from stage I–IV patients with ovarian cancer. DNA from tissue samples was extracted and analyzed by a pyrosequencing method following multiplex polymerase chain reaction. The genotypes of these SNPs were analyzed in the present study in a population of 105 patients, with different types of ovarian cancer, between 2004 and 2012. The allele frequencies for TLR4 Asp299Gly identified in this population were 1.00 (A) and 0.00 (G); for TLR4 Thr399Ile the allele frequencies were; 1.00 (C) and 0.00 (T). For TLR4 Asp299Gly the observed genotype frequency was 1.00 (AA), 0.00 (AG) and 0.0 (GG). In TLR4 Thr399Ile the observed genotype frequencies were 1.00 (CC), 0.00 (CT) and 0.00 (TT). TLR4 Asp299Gly and Thr399Ile alleles were not detected in the patients. These results indicated that the TLR4 299Gly and 399Ile alleles were exhibited at a lower frequency in the ovarian cancer patients that were examined.

## Introduction

Chronic infection and inflammation are important epigenetic factors contributing to tumorigenensis and tumor progession (1). Toll-like receptors (TLRs) are important pattern recognition receptors expressed by immune cells. Toll-like receptor 4 (TLR4) expression has been investigated in tumor cells or cell lines, including gastric carcinoma, extranodal marginal zone B-cell lymphomas, pituitary epithelial tumor cell lines, hepatocellular carcinoma cells, colon cancer cells and human prostate epithelial PC3 cells. Although TLR4 is expressed in numerous non-immune cells and tumor cells, the functional association of TLR4 with tumor progression requires further elucidation. The TLR4 single-nucleotide polymorphisms (SNPs), Asp299 and Thr399, have been reported to be involved in inflammation and cancer (2,3).

It was hypothesized that the presence of TLR4 variants may lead to the development of ovarian cancer. In the present study, the relevance of TLR4 SNPs, Asp299Gly (rs4986790) and Thr399Ile (rs4986791) are investigated in 105 ovarian cancer patients retrospectively with an extended follow-up and complete representative adjuvant therapy (chemotherapy with or without surgical treatment).

## Patients and methods

### Patients and tissue samples

Tissue specimens of 105 ovarian cancer patients (mean age, 54±11.7 years) were collected by the Department of Pathology, Shandong Cancer Hospital and Institute (Jinan, China). All patients were diagnosed and treated at the Department of Gynecologic Oncology, Shandong Cancer Hospital and Institute between 2004 and 2012. Treatment strategies were determined based on consensus recommendations from gynecological oncologists, which were based on guidelines for ovarian cancer treatment at the time (4). All patients provided written informed consent for the use of their tissues and participation in the study. The tissue samples were obtained during diagnostic or therapeutic surgery. Overall, 75 (71%) patients received paclitaxel + carboplatin chemotherapy prior or subsequent to surgery. A total of 19 (18%) patients received other chemotherapy prior or following surgery, and 11 (11%) patients received only surgery. Regular follow-up procedures were performed and the median follow-up in patients who had survived until the time of analysis was 26±17.6 months (range, 1–96 months).

### Sequence analysis of TLR4

As described in a previous study (5), DNA samples were extracted from 10-μm sections of formalin-fixed, paraffin-embedded tumor tissue. The germline mutations, TLR4 Asp299Gly (rs4986790) and Thr399Ile (rs4986791) were analyzed in all patients using pyrosequencing. For TLR4 Asp299Gly (rs4986790) and Thr399Ile (rs4986791), pyrosequencing was performed with the forward primer, 5′-TCTGGCTGGTTTAGAAGTCCA-3′; and the reverse primer, 5′-AATTGCCAGCCATTTTCAAG-3′; resulting in a 698-bp fragment. Following denaturation at 95°C, 35 cycles of DNA amplification were performed using Taq DNA Polymerase 2× Master Mix Red (Ampliqon-Biomol, Hamburg, Germany) at 95°C for 30 sec, 60°C for 30 sec and 72°C for 60 sec, with a final extension for 5 min at 72°C. Once the amplification was confirmed, the polymerase chain reaction product was digested for 1 h at 37°C with the restriction enzyme (Invitrogen Life Technologies, Carlsbad, CA, USA), *Nco*l (6). The SNP assays were purchased from Applied Biosystems, Inc., (ABI; Carlsbad, CA, USA) and performed on the ABI StepOnePlus^TM^ system. Data were analyzed using ABI StepOne^TM^ Software.

## Results

**Figure 1:**
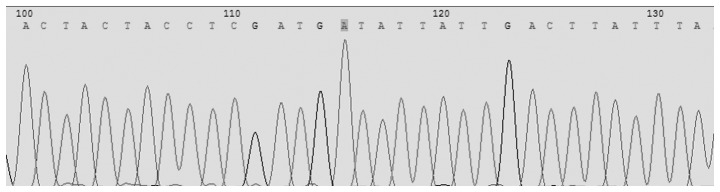
Sequence analysis of TLR4 Asp299Gly polymorphisms in ovarian cancer patients. The results of genoytype sequence CGATG**A**TATTA.

**Figure 2:**
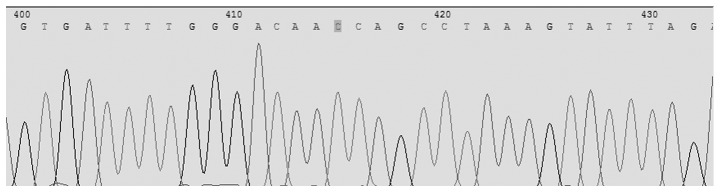
Sequence analysis of TLR4 Thr399Ile polymorphisms in ovarian cancer patients. The results of genoytype sequence GACAA**C**CAGCC.

Pyrosequencing was conducted for the simultaneous detection of Asp299Gly (rs4986790) and Thr399Ile (rs4986791) in the TLR4 gene. The allele frequencies for TLR4 Asp299Gly identified in this population were 1.00 (A) and 0.00 (G); for TLR4 Thr399Ile the allele frequencies were, 1.00 (C) and 0.00 (T). For TLR4 Asp299Gly the observed genotype frequency was 1.00 (AA), 0.00 (AG) and 0.0 (GG). In TLR4 Thr399Ile the observed genotype frequencies were 1.00 (CC), 0.00 (CT) and 0.00 (TT; Figs. 1 and 2).

## Discussion

Chronic inflammation is significant in the progression of various human cancers. Previous studies have revealed that inflammation-induced TLRs are involved in tumorigenesis (7–11). Additionally, certain studies have shown that the activation of TLR4 signaling may correlate with tumor progression (12). Thus, TLRs may be candidates as independent prognostic cancer markers (13).

The response to TLR ligands may be impaired by SNPs that are present in TLR genes, resulting in a modified susceptibility to the outcome of infectious or inflammatory diseases. Furthermore, the TLR genes have been shown to be polymorphic (6).

Currently, two SNPs within the human TLR4 gene have been focused on. The first is an A-G substitution at the 896-bp region, which results in an aspartic acid to glycine replacement at the 299 position of the amino acid sequence (Asp299Gly), and the other is a C-T substitution at the 1,196-bp region, which results in a threonine to isoleucine exchange at position 399 in the amino acid sequence (Thr399Ile) (2).

In breast cancer, data shows that patients who express the TLR4 loss of function allele experience a relapse more rapidly, following radiotherapy and chemotherapy, when compared with those patients who express the normal allele (14). For marginal zone B cells, TLR4 is the major receptor for lipopolysaccharide, and the rare TLR4 Asp299Gly allele attenuates the receptor signaling and decreases the inflammatory response. Data has shown that heterozygous genotypes are expressed significantly less frequently in patients with gastric mucosa-associated lymphoid tissue (MALT) lymphoma compared with that of *Helicobacter pylori*-infected control subjects. The TLR4 Asp299Gly genotype acts as a protective factor during the development of gastric MALT lymphoma in Caucasians (15).

Notably, there is an opposite conclusion for the TLR4 Asp299Gly/Thr399Ile polymorphism that is associated with gastric cancer. A study by Kato *et al* (16) reported no association between the TLR4 Asp299Gly polymorphism and gastric pre-cancerous lesions. However, de la Trejo *et al* (17) reported that the Asp299Gly polymorphism in TLR4 was significantly associated with duodenal ulcers and that there was a trend for an association with gastric cancer, with Asp299Gly polymorphism values similar in patients with or without the *H. pylori* infection. The Thr399Ile polymorphism in TLR4 was also identified as a genetic risk factor for gastritis and pre-cancerous lesions in a northern Indian population (18). Santini *et al* (19) demonstrated that the TLR4 Thr399Ile polymorphism is linked with an increased susceptibility to gastric cancer. However, other data indicates that TLR4 Asp299Gly and Thr399Ile are extremely rare in the Japanese population and, therefore, they may not be significant factors in establishing the outcome of *H. pylori*-infected Japanese patients (20). Guo *et al* (21) demonstrated that the polymorphism of cluster of differentiation, but not the TLR4 Asp299Gly mutation, was associated with a presence of colorectal cancer in Chinese patients. Zhang *et al* (22) indicated that using additional genetic models for rs4986790 and rs4986791 complicates analysis. Their meta-analysis indicated that the two SNPs (rs4986790 and rs4986791) in TLR4 were associated with an increased cancer risk, however, one SNP in TLR4 (rs1927911) was associated with a decreased cancer risk. Thus, the frequency of different polymorphisms has been shown to vary significantly across the different ethnic populations worldwide.

In conclusion, the TLR4 Asp299Gly and Thr399Ile alleles were not detected in the ovarian cancer patients in the present study. The results indicate that the TLR4 299Gly and 399Ile alleles have a markedly reduced frequency in northern Chinese ovarian cancer patients compared with those presented in the study by Zhang *et al* (21). Although detailed mechanisms and regulation of the TLR4 functions in tumor pathogenesis remain to be elucidated, TLR4 may be a promising target for the development of anticancer agents in the future.
